# Disparity Map Generation from Illumination Variant Stereo Images Using Efficient Hierarchical Dynamic Programming

**DOI:** 10.1155/2014/513417

**Published:** 2014-10-20

**Authors:** Viral H. Borisagar, Mukesh A. Zaveri

**Affiliations:** ^1^Computer Engineering Department, Government Engineering College, Gandhinagar, Gujarat 382028, India; ^2^Computer Engineering Department, Sardar Vallabhbhai National Institute of Technology, Surat, Gujarat 395007, India

## Abstract

A novel hierarchical stereo matching algorithm is presented which gives disparity map as output from illumination variant stereo pair. Illumination difference between two stereo images can lead to undesirable output. Stereo image pair often experience illumination variations due to many factors like real and practical situation, spatially and temporally separated camera positions, environmental illumination fluctuation, and the change in the strength or position of the light sources. Window matching and dynamic programming techniques are employed for disparity map estimation. Good quality disparity map is obtained with the optimized path. Homomorphic filtering is used as a preprocessing step to lessen illumination variation between the stereo images. Anisotropic diffusion is used to refine disparity map to give high quality disparity map as a final output. The robust performance of the proposed approach is suitable for real life circumstances where there will be always illumination variation between the images. The matching is carried out in a sequence of images representing the same scene, however in different resolutions. The hierarchical approach adopted decreases the computation time of the stereo matching problem. This algorithm can be helpful in applications like robot navigation, extraction of information from aerial surveys, 3D scene reconstruction, and military and security applications. Similarity measure SAD is often sensitive to illumination variation. It produces unacceptable disparity map results for illumination variant left and right images. Experimental results show that our proposed algorithm produces quality disparity maps for both wide range of illumination variant and invariant stereo image pair.

## 1. Introduction

Stereo vision is one of the challenging problems in computer vision. It infers 3D structure from images acquired with different viewpoints. Stereo vision has been researched widely due to its usefulness in many applications like 3D scene reconstruction, robot navigation, parts inspection, virtual reality, teleconferencing, extraction of information from aerial surveys, image-based rendering, and military and security applications. It can be divided into two problems: matching and 3D reconstruction. The most difficult problem in stereo vision is the establishment of visual correspondence among images. Most of the stereo matching algorithms assume illumination invariant stereo image pair. However there exist numerous real and difficult situations in which illumination variation between stereo images is unavoidable. There are few algorithms which reduce the effect of illumination variation between stereo pair to generate quality disparity maps. In [1] an extensive review of stereo matching algorithms is given. The difference in the positions of the two corresponding pixels in their respective images is called disparity. The corresponding pixels are the projections of a single pixel in the 3D scene. Two image pixels *q* and *q*_0_ match if they result from the projection of the same pixel *Q* in the scene.

In general, stereo algorithms can be classified into two major classes [1]: the methods based on local constraints and the methods based on global constraints. Local methods estimate disparity at a pixel in one image by comparing a small window about that pixel with a series of small windows extracted from the other image. Different types of windows, like solid windows [2], multiple windows [3, 4], and adaptive windows [5], have been used. Local method uses difference between color and intensity to find all the corresponding pixels in two or more images. It works on the assumption that neighboring pixels usually have the same disparity. This method gives good result in textured regions and faces many difficulties especially in texture less regions, disparity discontinuous boundaries, and occluded regions; algorithms solve these difficulties according to constraints [6]. Global methods make explicit smoothness assumptions and then solve it through various optimization methods. However, these methods have high computational complexity due to iterative approach for energy minimization and the complex constraints. Therefore, it is not suitable to apply these methods in real-time applications. These methods are less susceptible to ambiguous regions since global constraints provide additional support for regions which are difficult to match locally.

The appearance of corresponding pixels will vary in stereo images due to different perspective projections. Pixel in one image may have many matching pixels in the second image especially in repetitive texture or textureless areas. This is called the problem of ambiguity. There are many constraints that make stereo an ill-posed problem. One type of issue is associated with the scene itself, specifically textureless areas, non-Lambertian surfaces, reflections, translucency, and occlusions. The others are camera-related issues like image noise, errors due to imperfect calibration, and orientation of camera. The utilization of multiple cameras to capture images introduces problems such as differences in exposure, white balancing, and other radiometric properties. Stereo algorithms should be reliable and efficient to be useful in real-time applications.

Nonuniform illumination between two images frequently occurs in real scenario. Stereo algorithms should be capable to handle such type of difficult illumination conditions [7]. Even with close to ideal measurements and least illumination deviation between images, the matching is still ambiguous for a large number of pixels. Mostly, illumination conditions of two consecutive images are nearly the same. Even though, a quick change of illumination might occur within short time. Illumination normalization [8] is essential to the stereo matching algorithms sensitive to illumination variation.

Similarity measures such as sum of absolute difference (SAD) and absolute difference (AD) [1] are used to calculate the matching costs to find the corresponding pixels. These methods suppose that matching pixels between left and right images will have the same color value. But due to illumination variation the corresponding pixels will have different color values [9]. Thus in case of illumination variation the estimated disparity map is extensively degraded as compared to the ground truth.

Dynamic programming (DP) named by Richard Bellman [10] in 1953 is an effective global method to compute correspondences for pixels of an image. The minimum cost path going down and right from the top-left corner of the grid to its bottom-right corner is found. Thus, dynamic programming method can be used to find the best possible match sequence between the start and end points.

Many researchers have been attracted towards global algorithms due to their accurate results. Global algorithms such as graph cut [11–13] and belief propagation [14–16] give good results by finding local minima, that is, minima over large neighborhood. As these methods are computationally very costly they are not suitable for real-time applications. Since many years a lot of research is going on to develop real-time algorithms for disparity estimation. Global optimization techniques based on dynamic programming (DP) are gradually becoming acceptable in such type of algorithms because of its high effectiveness [17–19]. For all scanlines optimization in the image is performed independently in dynamic programming. However, this method needs strict enforcement of ordering constraints [20]. It provides global solution for the disparity estimation under local constraints like correlation, smoothness, and disparity gradient limit. It produces quality results on occlusion boundaries but ‘‘streaking” artifact is produced because of the inconsistency between scanlines.

The assumption that a 3D point in space has the same appearance under 2D projection from different geometries is not always true in case of illumination variation. Corresponding pixels in left and right images will have different color values due to different illumination conditions. Stereo image pair acquired by the camera often experience illumination variations due to spatially and temporally separated camera positions. Illumination variation between left and right image occurs because of varying lighting and exposure differences. The scene is assumed to be Lambertian in most of the disparity generation algorithms. This assumption is violated in real scenario due to the existence of illumination variation. When images of a stationary scene are recorded by camera at different timing interval, illumination variation occurs due to environmental illumination fluctuation and the change in the strength or position of the light sources. It may not be possible to manage the light source, for example, outdoors when image acquisition of large scenes will take some time. Such scenario exists during matching of aerial or satellite images. Because of all of the above mentioned factors, it can be concluded that real world stereo imaging algorithms should have illumination variation robustness.

The scope of this paper is to generate disparity map from a wide variety of illumination variant stereo image pair. Most of the stereo matching algorithms assume illumination invariant stereo image pair. However there exist numerous real and difficult situations in which illumination variation between stereo images is unavoidable. There are few algorithms which reduce the effect of illumination variation between stereo pair to generate quality disparity maps. It is not a trivial problem to estimate the disparity map from the stereo image pair having illumination variation as it seriously influences the performance of stereo matching algorithms. In order to get illumination variant stereo images changes are made synthetically in the Middlebury Stereo Datasets [1, 21]. Matching stereo image pair acquired under different illumination increases the error. Simulation results demonstrate that the proposed algorithm generates quality disparity maps if portion of one of the images of stereo pair is changed due to illumination. Our algorithm gives quality results for both illumination variant and invariant stereo image pair.

The rest of the paper is organized as follows: in Section 2 related work is explained and then in Section 3 proposed hierarchical stereo matching algorithm is described in detail.  Section 4 discusses experimental results and demonstrates the performance of our algorithm.  Section 5 depicts the conclusion of this paper and future work. At the end, references are listed.

## 2. Related Work

A stereo algorithm [22] combines the use of lightness-invariant pixel difference evaluation within a dynamic programming depth estimation approach. This algorithm uses dynamic programming technique, projection of the HSL color space for lightness tolerance, and gestalt-based adaptive support weight aggregation. By applying histogram equalization to each color channel illumination normalization is done. The drawbacks of histogram equalization are color distortion and noise amplification [23]. To decrease the effect of these drawbacks, an illumination normalization technique is proposed in [8] in which the histogram of the second image is adjusted in accordance with that of the first image. Baker and Binford [24] worked on stereo using dynamic programming. Each probable solution of the disparity field is represented in the form of path through matching space. The monotonicity confines the space of probable solutions.

The effect of illumination variation can be reduced by two main approaches. Firstly, many algorithms are proposed to lessen the effect of illumination variations by normalizing color values [25–27]. Such algorithms are applied as a preprocessing step before the stereo matching to reduce the effect of illumination variation. Colors are normalized based on the averages by the grey-world assumption algorithm [25]. The dependency on the effect of global illumination and scene geometry is tried to be eradicated iteratively by the comprehensive color normalization algorithm [26]. The grey-edge algorithm [27] utilizes the average edge difference for the color normalization. Secondly, some algorithms utilize color invariant similarity measures [28, 29] for matching cost calculation. Similarity measure normalized cross-correlation (NCC) reduces the effect of illumination variation by compensating the gain and the bias difference between stereo images [28]. The adaptive normalized cross-correlation (ANCC) [29] utilizes the NCC adaptively based on the comprehensive color normalization.

The global minimum for independent scanlines can be found in polynomial time by using dynamic programming. In stereo vision DP was initially used in sparse, edge-based techniques [24, 30]. Dense (intensity-based) scanline optimization problem [31, 32] is addressed by current approaches. Minimum cost path through the matrix of all pairwise matching costs between two corresponding scanlines is calculated by these approaches.

Dynamic programming (DP) is used for matching epipolar scanlines and then the solutions are improved iteratively using edges [30]. Stereo matching algorithm based on DP considering the interscanline constraint is discussed in [32]. This technique needs less number of iterations to estimate the global solution. Bayesian approach for stereo matching is discussed in [31]. First of all DP is applied along epipolar scanlines to obtain initial estimate of disparity values and thereafter vertical smoothing is performed iteratively. To decrease the inconsistency among scanlines [33, 34] few DP based approaches use the concept of ground control points or reliability-based matching cost computation and few use the approach of global optimization [18, 34].

In horizontal grid type the smoothness constraints are imposed only within the horizontal scanlines. In [35] the optimization of the energy function for each scanline is done separately by using DP. Inconsistency between scanlines, that is, streaking effect, is produced in the output due to skipping of vertical edges. Graph-based global methods are proposed to solve this problem of streaking effect. To optimize this problem approaches like graph cuts [11] and belief propagation [36] can be used. These approaches are giving quality output but are computationally very expensive.

Multiresolution stereo matching exists in addition to cost calculation for matching and disparity optimization. This hierarchical stereo matching method can be implemented using image pyramids to improve the disparity estimation based on the coarse-to-fine approach [37, 38]. Multiresolution or coarse-to-fine paradigm for stereo matching is faster than one without multiresolution [39], because the search range in each stage of the pyramid is small. In addition to fast computation, higher quality disparity map can also be obtained by utilizing the multiresolution data structure. Overview of the scene can be obtained at coarse level and detailed information can be found at fine level. There are three advantages of coarse-to-fine method [40]: (a) search range can be increased, because at a lower resolution pyramidal level only rough initial values are required; (b) increase in speed can be achieved as only the neighborhood of the previous result requires to be searched; and (c) the reliability of finding the right matching can be increased.

The assignment of the accurate cost for occluded pixels and maintenance of interscanline consistency are the difficulties associated with the dynamic programming. Various algorithms are proposed to address interscanline consistency [30–32]. The monotonicity or ordering constraint [20] is needed to be imposed in dynamic programming. The relative ordering of pixels on a scanline must be the same between the two views in this constraint, which may not be the case for thin foreground objects containing images.

Anisotropic diffusion has been applied as the regularization term by latest energy-based methods to avoid oversmoothing of significant edges. Perona and Malik [41] proposed anisotropic diffusion to achieve image enhancement and restoration. The diffusion coefficient is changed at edges with sharp intensity gradients. This technique is used for disparity and depth generation.

## 3. Proposed Algorithm

Many stereo matching algorithms assume illumination invariant stereo image pair. However there exist numerous real and difficult situations in which illumination variation between stereo images is unavoidable. When corresponding pixels between left and right images have dissimilar colors because of illumination variations as shown in Figures 4(a) and 4(b), the inaccurate disparity map is generated. A new hierarchical stereo vision algorithm is proposed which gives disparity map as output from illumination variant stereo pair. Illumination variation between two stereo images affects the quality of the disparity map generated. When images of a stationary scene are recorded by camera at different timing interval, illumination variation occurs due to environmental illumination fluctuation and the change in the strength or position of the light sources. Hence there will be illumination variation between left and right images in many real-time situations. This illumination variation degrades the performance of stereo matching algorithms. If illumination variation is not suppressed in preprocessing stage, then that will lead to false matching and in turn will result into inaccurate disparity map estimation. So in order to get accurate disparity map as output, illumination difference between two images must be suppressed before starting matching process. Illumination variation suppression is done by using homomorphic filtering to make the pixels of both images more or less illumination invariant.

Homomorphic filtering of both images will improve the quality of input images. It is a general method for image and signal processing. First of all nonlinear mapping to a different domain is done and then linear filtering methods are applied on the image. Lastly mapping back to the original domain is performed. This concept was developed in the 1960s by Thomas Stockham, Alan V. Oppenheim, and Ronald W. Schafer at MIT [42]. This filtering method has been used in various imaging applications such as robotics, biometrics, and image enhancement.

Image can be viewed as a product of illumination and reflectance of scene. The low frequency components due to illumination can be removed by taking the log of the image and thereafter applying high-pass filtering. In this way, enhancement of details within an image is done by homomorphic filtering.

Illumination variations can be treated as a multiplicative noise and can be reduced by filtering in the log domain. The brightness across an image is normalized by homomorphic filtering and it also enhances contrast. Multiplicative noise can be removed by using homomorphic filtering. Approximate position of illumination and reflectance can be found in frequency domain. Illumination and reflectance are combined together multiplicatively. These multiplicative components of the image are made additive and thus can be separated linearly in the frequency domain by taking the logarithm of the image intensity.

The reflectance is represented by the high frequency components whereas illumination in the scene is represented by the low frequency components. The high frequency components are increased and low frequency components are decreased to normalize the illumination of an image. Thus in the log-intensity domain [43], suppression of low frequencies and amplification of high frequencies are done by high-pass filtering.

Homomorphic filtering method uses illumination-reflectance model. Image can be characterized by two major components in this model. The first component is the amount of source illumination *i*(*x*, *y*) incident on the scene being viewed and reflectance component *r*(*x*, *y*) of the objects in the scene is the second component. The image *f*(*x*, *y*) can be related [44–46] to *i*(*x*, *y*) and *r*(*x*, *y*) as mentioned below:

(1) $$\begin{array}{c} f\left(x,y\right)=i\left(x,y\right)r\left(x,y\right). \end{array}$$

Thus, according to (1) intensity at a particular pixel within an image is a product of illumination and reflectance. As intensity *i*(*x*, *y*) alters slowly compared to *r*(*x*, *y*), *i*(*x*, *y*) is having more number of low frequency components than *r*(*x*, *y*). *r*(*x*, *y*) leads to high frequency components mainly at the borders of two reflecting objects. The main purpose of homomorphic filtering is to lessen the effect of *i*(*x*, *y*) by decreasing the low frequency components of the image and then filtering process is performed. Homomorphic filtering can be implemented using the following steps [46].

**Step 1 .**

Take a natural logarithm on both sides of (1) to change the multiplication operation between *i*(*x*, *y*) and *r*(*x*, *y*) into addition operation:

(2) $$\begin{array}{c} z\left(x,y\right)=\ln i\left(x,y\right)+\ln r\left(x,y\right). \end{array}$$

**Step 2 .**

Image is transformed from spatial domain to frequency domain by using Fourier transform

(3) $$\begin{array}{c} I\left\{z\left(x,y\right)\right\}=I\left\{\ln i\left(x,y\right)\right\}+I\left\{\ln r\left(x,y\right)\right\} \end{array}$$

or

(4) $$\begin{array}{c} Z\left(u,v\right)=F_{i}\left(u,v\right)+F_{r}\left(u,v\right), \end{array}$$

where *F*_*i*_(*u*, *v*) and *F*_*r*_(*u*, *v*) are the Fourier transforms of ln⁡*i*(*x*, *y*) and ln⁡*r*(*x*, *y*), respectively.

**Step 3 .**

Filter function *H*(*u*, *v*) is used to high pass the *Z*(*u*, *v*) to get a filtered *S*(*u*, *v*):

(5) S(u,v)=H(u,v)Z(u,v)=H(u,v)Fi(u,v)+H(u,v)Fr(u,v).

**Step 4 .**

To get the filtered image in spatial domain, an inverse Fourier transformation is applied:

(6) s(x,y)=I−1{S(u,v)}=I−1{H(u,v)Fi(u,v)+H(u,v)Fr(u,v)},s(x,y)=i′(x,y)+r′(x,y).

**Step 5 .**

The filtered enhanced image *j*(*x*, *y*) can be obtained as follows:

(7) $$\begin{array}{c} j\left(x,y\right)=\exp\left\{s\left(x,y\right)\right\},\\ j\left(x,y\right)=\exp\left\{i^{\prime }\left(x,y\right)\right\}\cdot \exp\left\{r^{\prime }\left(x,y\right)\right\},\\ j\left(x,y\right)=i_{0}\left(x,y\right)r_{0}\left(x,y\right), \end{array}$$

where *i*_0_(*x*, *y*) and *r*_0_(*x*, *y*) are the illumination and reflectance components, respectively.

In this way, low frequency components can be reduced and high frequency components can be improved. Homomorphic filter decreases the contribution due to illumination and information which is not available in the original image can be seen in the enhanced image. Hierarchical dynamic programming is applied on both left and right images after illumination variation suppression. It minimizes the global cost for each scanline of image to determine the optimal path, that is, least costly disparity values for each pixel.

In dynamic programming (DP) the problem is divided into smaller problems recursively; the smallest problem is solved first and its answer is used to solve the problem of the preceding level [47]. In case of stereo, the problem is to find the best path of matching between two scanlines, that is, to find the disparity field with minimum cost. Error propagation issues on the same line can be avoided by solving every small problem globally. DP is proficient algorithm to solve sequential decision, that is, optimal path problems.

DP stereo algorithm treats the correspondence problem as an energy minimization problem. The energy function *E*(*d*) denotes the total cost of a sequence of matching pixels in a scanline. According to (8), *E*(*d*) consists of a data and smoothness term:

(8) $$\begin{array}{c} E\left(d\right)=E_{d}\left(d\right)+⋋E_{s}\left(d\right), \end{array}$$

where *E*_*d*_(*d*) denotes matching cost. For every pixel, it is used to find the best matching pixel in the other image. *E*_*s*_(*d*) indicates smoothness cost, which ensures that neighboring pixels should usually have same disparity.

DP gives better disparity map compared to that obtained by correlation methods. It is also speedy compared to correlation and requires lesser scanline buffers than correlation window. It moves forward in a scanline, produces solution by backtracking through predecessor array, averages noise over a scanline, does not alter values in backtrack, identifies occlusions, and utilizes ordering constraint to give global solution along scanlines. If a pixel is on the right of a matched pixel in the left scanline, then its corresponding pixel will also be on the right of the matched pixel in the right scanline.

**Figure 1:**
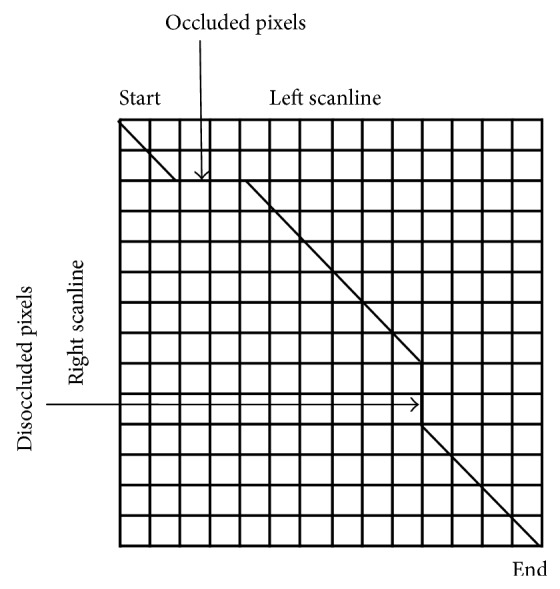
Dynamic programming for stereo matching.

DP gives the optimal path through grid as shown in Figure 1. This path gives the best set of matches satisfying the ordering constraint. In DP raster intensities are arranged on two sides of a grid. The diagonal line within grid indicates possible correspondences. Optimal cost for each node is calculated from given upper-left neighbors. In order to find the minimum cost path, the least cost path for every pixel in a scanline is computed starting from the origin, left to right and top to bottom. Disparity of each pixel can be calculated by path backtracking. A cost is associated with every path and out of all paths the optimal one is selected.

As shown in Figure 1, pixel can be either sequential or occluded or disoccluded. The cost of matching has to be calculated for sequential pixels whereas it will not be computed for occluded and disoccluded pixels.

The typical implementation of DP [48] can be described as follows.

**Step 1 .**

Initialization:

(9) $$\begin{array}{c} \alpha \left(i,d\right)=\text{cost}\left(i,d\right),\;i=0,\;\;\min D\leq d\leq \max D\\ \text{path}\left(i,d\right)=0. \end{array}$$

**Step 2 .**

Recursion from time *i* = 1 to *S* − 1:

(10) $$\begin{array}{c} \alpha \left(i,d\right)=\underset{k}{\min}\left\{\alpha \left(i-1,k\right)+\text{cost}\left(i,d\right)+P\left(d-k\right)\right\},\\ \min D\leq d,\;\;k\leq \max D\\ \text{path}\left(i,d\right)=\arg\underset{k}{\min}\left\{\alpha \left(i-1,k\right)+\text{cost}\left(i,d\right)+P\left(d-k\right)\right\}. \end{array}$$

**Step 3 .**

Termination:

(11) $$\begin{array}{c} {\;d}_{S-1}=\arg\underset{d}{\min}\left\{\alpha \left(L-1,d\right)\right\}. \end{array}$$

**Step 4 .**

Path backtracking from time *i* = *L* − 2 to 0:

(12) $$\begin{array}{c} d_{i}=\text{path}\left(i+1,d_{i+1}\right), \end{array}$$

where cost(*i*, *d*) is the matching cost, *L* is the length of a scanline, *P*(*d* − *k*) is the penalty for inconsistent disparity, *i* is the point index of the second scanline, *α*(*i*, *d*) is the accumulated matching cost at *i*th pixel with a disparity *d*, min⁡*D* and max⁡*D* are the minimum disparity and the maximum disparity, respectively, and *d*_*i*_ is the disparity of the pixel *i* estimated by the DP.

We provide preprocessed color stereo image pair as input and convert the images to gray scale for the matching process and perform window matching. As the images are rectified, search has to be done only over columns and not over rows. Window matching estimates the disparity for every pixel based on its cost function. The disparity values obtained by window matching are all integers; the disparity map obtained displays contouring effect as there is no smooth transition between areas having different disparity. This problem of contouring effect can be solved by introducing subpixel calculation into the matching metric. Fast method of interpolation is required to increase the speed. Every pixel *L*_*xy*_ of left image is compared with *R*_*x*(*y*−1−*d*)_, *R*_*x*(*y*−*d*)_, and *R*_*x*(*y*+1−*d*)_ pixels of the right image to find the dissimilarity, where *d* is the disparity obtained by the stereo algorithm. A quadratic polynomial is then fitted to these three dissimilarity values obtained and its minimum is found which is the refined floating point disparity value associated with pixel *L*_*xy*_. This disparity value may lie to the right or left of *y*. In textureless areas, the minimum value may not lie in between *d* − 1 and *d* + 1. In such conditions, depending on where the dissimilarity value is the least either *d* − 1 or *d* + 1 is selected. Thus by introducing subpixel computation with window matching contouring effects are mostly removed and refined values of disparity are obtained.

Now we want to assign disparity value with probably suboptimal cost to pixel locally. Increasing pixel's agreement in disparity with its neighbors will balance this additional cost. Each disparity estimate is confined to lie near to disparity values of its neighboring pixels along an image row. The problem of estimating the best possible disparity values for a row of pixels is now converted into the problem of finding the optimal path from top-left part to bottom-right part of the image. Metric obtained after subpixel calculation is used as the cost function and disparity values are allowed to change only by a specific amount between neighboring pixels to find the optimal path. We have attempted to solve this problem by using dynamic programming.

Dynamic programming increases the correctness of the disparity map. However the computational complexity is increased due to both window matching and dynamic programming. Hierarchical DP can be used along with the window matching [49, 50] to solve this problem of computational complexity. Hierarchical DP, as originally proposed in [51], works on almost the same principles as of typical DP. This hierarchical stereo matching method is implemented using image pyramids to improve the disparity estimates based on the coarse-to-fine model. The images are downsampled an optimal number of times and the disparity map of a coarser level is used as an “offset” disparity map at a finer level. Here we have considered image pyramids with 3 levels for the coarse-to-fine process. For downsampling a 2D image, the image is subdivided into groups of 4 pixels and its average is calculated. In the hierarchical approach, standard DP is performed on the lowest level and the results obtained are used as an offset for the next higher level to find the minimum cost. Thus search over a smaller range of disparities is required. This process is continued until the highest level, that is, original image, is reached. In the original image search over a 15 pixel range is required to estimate the disparities. If the image is downsized by a factor of two, this search reduces to 7 pixels on an image with a quarter of the area; this approach would cost less by a factor of 8. The results obtained by this hierarchical approach are of high quality and are achieved at a reduced computational cost compared to combination of both window matching and dynamic programming.

In DP algorithm individual scanlines are matched well but there is no intrascanline consistency. Scanlines in the reference image are matched independent of each other in DP and the output contains disparity discontinuities in the vertical direction of the image, giving a streaky appearance. The hierarchical DP gives better results because of the inherent image smoothing resulting from downsampling. But if scanline inconsistency is made on lower level of the hierarchy, that error is propagated all the way to the top level.

The result obtained from hierarchical DP is smoothed by applying anisotropic diffusion [41, 52] instead of plane fitting. Diffusion is a physical process which plans to minimize differences in the spatial concentration *u*(*x*, *t*) of a substance. The aim is to have less diffusion, that is, smoothing on edge locations. Anisotropic diffusion is a smoothing method that smoothes images without crossing any edges. Anisotropic diffusion conserves and increases the contrast at sharp intensity gradients. Gradient norm $\left|\nabla u\right|=\sqrt{{u_{x}}^{2}+{u_{y}}^{2}}$ serves as edge indicator. Diffusivity should decrease with increasing |∇*u*|. The smoothing mechanism follows the following diffusion equation:

(13) $$\begin{array}{c} g\left(\left|\nabla u\right|\right)=\frac{1}{\sqrt{1+{\left|\nabla u\right|}^{2}/\lambda ²}}. \end{array}$$

*λ* > 0 is called a contrast parameter. Areas where |∇*u*| ≫ *λ* will not be affected much by the diffusion process.

Thus a refined disparity map without blurring any edges is obtained.

## 4. Results and Discussion

In this section, we present the experimental results of our algorithm. The Middlebury Dataset [1, 21] is used to evaluate the results of the proposed algorithm. The popular and extensively used image pairs like tsukuba, cones, venus, and sawtooth are used for an evaluation purpose. These images contain different objects with different characteristics. Computation of our proposed algorithm is carried out in Matlab version: 7.14.0.739 (R2012a) on Intel(R) Core(TM) i3 CPU M 350 @ 2.27 GHz (4 CPUs) laptop.

The proposed algorithm has been applied to a series of illumination variant stereo pairs. As shown in Figure 4, our proposed algorithm is tested by using the tsukuba image pair having 0% (first row), 6.25% (second row), 12.50% (third row), 25% (fourth row), and 50% (fifth row) pixel illumination variation between left and right images. All these images are derived by changing the values of each one of the RGB channels of left tsukuba image by 10% and keeping right tsukuba image unchanged. Thus a series of tsukuba pairs is obtained with a fixed illumination difference between the left and right image of each pair. The proposed algorithm is tested on these image pairs and quality results are obtained as shown in Figure 4.

To evaluate the performance of a stereo algorithm we need a quantitative way to estimate the quality of the computed disparity map. The quality of the estimated disparity map can be compared with respect to ground truth by using similarity measure root mean square error. RMSE is calculated in terms of disparity units between the estimated disparity map *d*_*C*_(*x*, *y*) and the ground truth *d*_*T*_(*x*, *y*) and is given as follows:

(14) $$\begin{array}{c} R=\sqrt{\left(\frac{1}{N}\;\underset{\left(x,y\right)}{\sum }{\left|d_{C}\left(x,y\right)-d_{T}\left(x,y\right)\right|}^{2}\right)}, \end{array}$$

where *N* is the total number of pixels.

Table 1 shows the root mean square error (RMSE) of the disparity maps generated for each of the tsukuba pairs shown in Figure 4 with respect to the ground truth disparity maps. It also shows comparison of results of our proposed algorithm based on hierarchical DP with the results obtained by similarity measure sum of absolute difference (SAD). RMSE values are obtained by comparing estimated disparity map with the ground truth of the corresponding image. Here, in our proposed approach based on hierarchical DP we have considered image pyramids with 3 levels and disparity search range of 4 and size of the tsukuba image is 384 × 288. Results of SAD are directly obtained by giving different illumination variant tsukuba image pairs as input without applying any preprocessing and postprocessing steps. Figures 3 and 4 and Table 1 demonstrate that in case of illumination variant images SAD gives undesirable results, while our proposed approach gives excellent results.

The sum of absolute differences (SAD) does not consider varying illumination conditions.  Figure 3(d) shows that inaccurate disparity map is estimated by using similarity measure SAD by giving tsukuba stereo image pair as input. 50% of total pixels of input left image are changed due to 10% illumination variation and the pixels of right image are left unchanged. As the illumination variation increases, the quality of estimated disparity map is affected which leads to increase in RMSE values. Figures 3(d) and 4(c) (fifth row) and Table 1 show that the performance of our proposed algorithm is better than the SAD method.

The performance of the proposed algorithm is summarized and compared with the results obtained by conventional image pyramiding approach in Table 2. Results of image pyramiding approach are obtained without applying homomorphic filtering, dynamic programming approach, and anisotropic diffusion. Image pyramiding stereo matching is applied to calculate the matching cost from coarse level to fine level. Image pyramiding or coarse-to-fine approach for stereo matching is faster than one without image pyramiding, because the search range in each stage of the pyramid is small. Outline of the scene can be obtained at coarse level and thorough information can be found at fine level. We have applied window based method SAD at each level to calculate the matching cost. As the results of coarser level are inaccurate due to the presence of illumination variation, it also affects the results of finer level leading to generation of inaccurate disparity map. Table 2 shows the comparison between root mean square error (RMSE) values obtained for our proposed approach based on hierarchical DP and image pyramiding approach.

**Figure 2:**
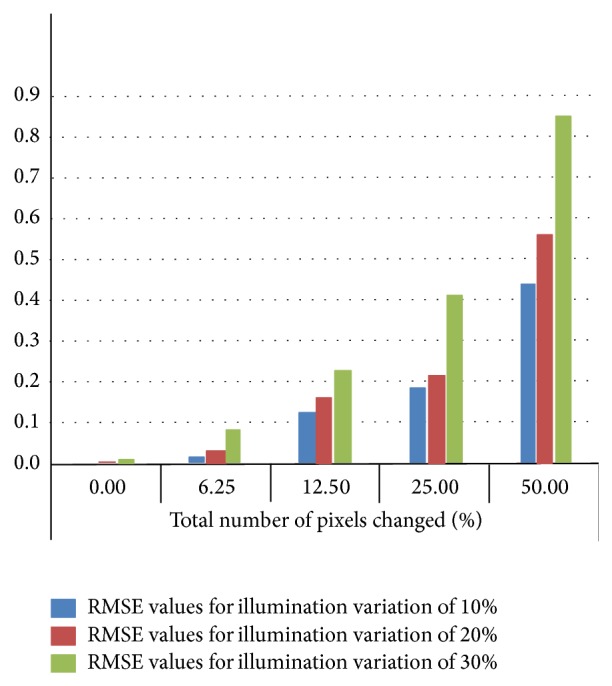
Comparison of RMSE values obtained under different illumination variation with different percentage of pixels within image changed (input stereo pair: tsukuba).

Figure 2 shows the comparison of RMSE values obtained under 10%, 20%, and 30% illumination variation between tsukuba image pair with 0%, 6.25%, 12.50%, 25%, and 50% of total pixels changed within image due to illumination variation. As the illumination variation increases the quality of estimated disparity map is affected which leads to increase in RMSE values. Also the increase in number of affected pixels in an image due to illumination variation leads to increase in RMSE values.

Table 3 compares RMSE values obtained for illumination variant stereo pair like venus, cones, and sawtooth as input to our proposed algorithm. These illumination variant images are generated synthetically by changing the values of each one of the RGB channels of left image by 10% and keeping right image unchanged. Our proposed algorithm is tested by using the venus, cones, and sawtooth image pair having 0%, 6.25%, 12.50%, 25%, and 50% pixel illumination variation between left and right images. From Table 3 it can be understood that our algorithm gives quality results for different types of stereo images having different image characteristics.

**Figure 3:**
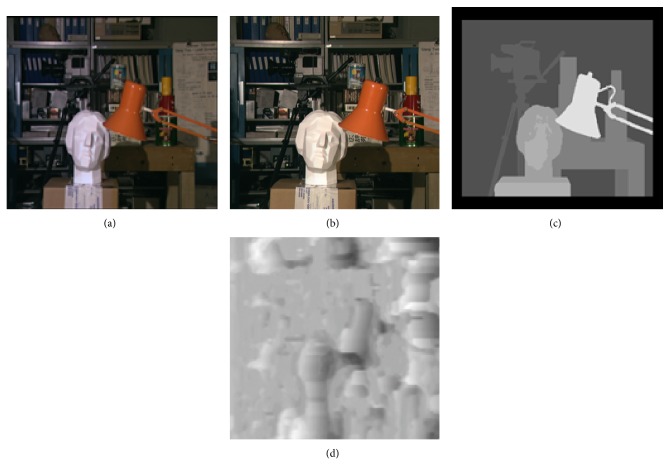
The disparity map generated by using conventional SAD for tsukuba stereo image pair with 50% of total pixels changed within image due to 10% illumination variation. (a) and (b) The left and right tsukuba image with varying illumination. (c) The ground truth. (d) The disparity map generated using SAD method for images (a) and (b).

**Figure 4:**
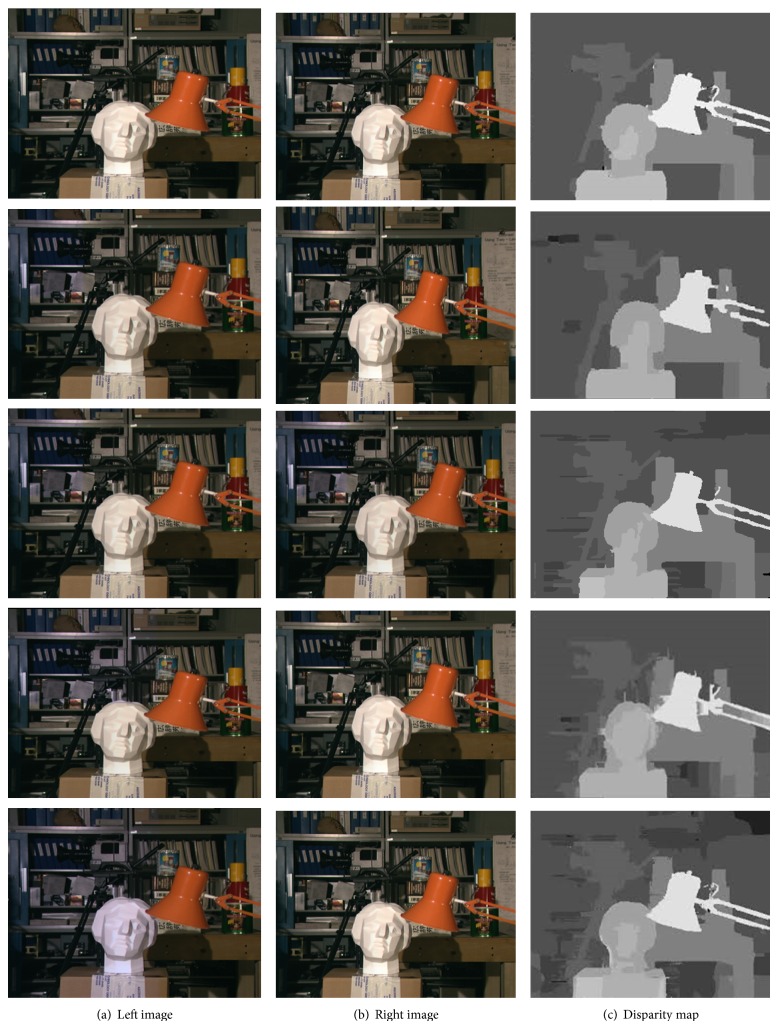
Disparity maps generated by our proposed algorithm for the Tsukuba dataset under 0% (first row), 6.25 % (second row), 12.50% (third row), 25% (fourth row), and 50% (fifth row) pixel illumination variation.

From the results shown in Figures 2, 3, and 4 and Tables 1, 2, and 3 it can be demonstrated that the output of our algorithm is mostly independent of the illumination variation between the two input stereo images. The proposed algorithm presents a robust behavior having low RMSE constantly over a wide range of illumination variation between the two input stereo images. The performance of our algorithm is comparable to the state-of-the-art stereo algorithms. The disparity maps estimated by our algorithm are of good quality. The background small objects in tsukuba image are not clearly visible in the estimated disparity map. The simulation results indicate that the proposed algorithm is efficient for disparity map generation under varying illumination conditions.

The quality of disparity map generated depends on the number of illumination sources used, percentage by which pixel value is changed due to illumination, percentage of pixels changed due to illumination, and characteristic of stereo images.

## 5. Conclusion and Future Work

This paper presents a novel, robust, efficient, and flexible hierarchical dynamic programming based stereo matching algorithm. Most of the stereo matching algorithms assume illumination invariant stereo image pair. However there exist numerous real and difficult situations in which illumination variation between stereo images is unavoidable. The algorithm presented can be useful in real-time application where there is illumination variation between left and right images.

We compared results of our proposed approach with the results of similarity measure SAD method. SAD method gives fast but inaccurate disparity map results, as it lacks the ability to lessen the effect of illumination variation. Window matching and DP are used for the generation of disparity map. The proposed approach preserves edge information very well. The hierarchical DP approach leads to reduction in the computational complexity compared to DP. But the computational complexity of hierarchical DP is more compared to image pyramiding approach. The total runtime for our proposed algorithm is 223.37 seconds and for image pyramiding the total time required is 112.23 seconds.

Evaluation and comparison of the root mean square error values for various illumination variations for different input stereo images like venus, cones, and sawtooth show that our proposed algorithm gives quality results for different stereo images having different image characteristics. Experiments on various standard datasets show that the performance of our algorithm is comparable to the state-of-the-art stereo algorithms. Testing on different stereo pairs reveals that homomorphic filtering deals with shot noise remarkably but result is affected due to the presence of Gaussian noise and strong brightness changes. Dynamic programming performs relatively well at low contrast regions. DP blurs the edges due to smoothness constraint and it also does nothing to smooth between rows. In spite of these drawbacks, the quality of the disparity map is improved, as the noise is almost completely removed and many of the objects are better reconstructed. Using hierarchical DP we have been able to efficiently find a minimum cost path.

Anisotropic diffusion method is applied instead of plane fitting for disparity map refinement. It is applied on the disparity map obtained by hierarchical DP for smoothing the disparity map along with preserving the edges. Anisotropic diffusion is an image smoothing method that smoothes images without crossing edges, while enhancing and conserving the contrast at sharp intensity gradients. The results obtained are of good quality. The background tiny objects present in tsukuba image are not clearly detectable in the generated disparity map. Simulation results demonstrate that the proposed algorithm generates quality disparity maps if portion of one of the images of stereo pair is changed due to illumination. Our algorithm gives quality results for both illumination variant and invariant stereo image pair. There is still a possibility to decrease the computational complexity. The accuracy of disparity map estimated depends on the number of illumination sources used, percentage by which pixel value is altered due to illumination, percentage of pixels changed due to illumination, and characteristic of stereo images. The proposed algorithm can be implemented using FPGA or GPU for hardware acceleration in future.

## Figures and Tables

**Table 1:** RMSE for various illumination variations (input stereo pair: tsukuba).

Sr. number	Illumination variation	RMSE (our proposed approach)	RMSE (SAD)
1	0.00%	0.004	24.34
2	6.25%	0.015	28.98
3	12.50%	0.128	39.48
4	25.00%	0.190	44.87
5	50.00%	0.440	47.22

**Table 2:** RMSE for various illumination variations (input stereo pair: tsukuba).

Sr. number	Illumination variation	RMSE (our proposed approach)	RMSE (image pyramiding)
1	0.00%	0.004	173.41
2	6.25%	0.015	258.32
3	12.50%	0.128	370.67
4	25.00%	0.190	523.59
5	50.00%	0.440	897.84

**Table 3:** RMSE for various illumination variations for different input stereo images.

Sr. number	Illumination variation	RMSE for stereo pair venus as input	RMSE for stereo pair cones as input	RMSE for stereo pair sawtooth as input
1	0.00%	0.018	0.039	0.00245
2	6.25%	0.027	0.085	0.04420
3	12.50%	0.149	0.343	0.20300
4	25.00%	0.230	0.548	0.45800
5	50.00%	0.653	0.872	0.97100
